# Polyamine-Derived Aminoaldehydes and Acrolein: Cytotoxicity, Reactivity and Analysis of the Induced Protein Modifications

**DOI:** 10.3390/molecules28217429

**Published:** 2023-11-04

**Authors:** Marek Šebela, Michaela Rašková

**Affiliations:** Department of Biochemistry, Faculty of Science, Palacký University, Šlechtitelů 27, 783 71 Olomouc, Czech Republic

**Keywords:** acrolein, aldehyde dehydrogenase, amine oxidase, aminoaldehyde, 3-aminopropanal, cytotoxicity, glutathione, Michael adduct, protein modification, Schiff base

## Abstract

Polyamines participate in the processes of cell growth and development. The degradation branch of their metabolism involves amine oxidases. The oxidation of spermine, spermidine and putrescine releases hydrogen peroxide and the corresponding aminoaldehyde. Polyamine-derived aminoaldehydes have been found to be cytotoxic, and they represent the subject of this review. 3-aminopropanal disrupts the lysosomal membrane and triggers apoptosis or necrosis in the damaged cells. It is implicated in the pathogenesis of cerebral ischemia. Furthermore, 3-aminopropanal yields acrolein through the elimination of ammonia. This reactive aldehyde is also generated by the decomposition of aminoaldehydes produced in the reaction of serum amine oxidase with spermidine or spermine. In addition, acrolein is a common environmental pollutant. It causes covalent modifications of proteins, including carbonylation, the production of Michael-type adducts and cross-linking, and it has been associated with inflammation-related diseases. APAL and acrolein are detoxified by aldehyde dehydrogenases and other mechanisms. High-performance liquid chromatography, immunochemistry and mass spectrometry have been largely used to analyze the presence of polyamine-derived aminoaldehydes and protein modifications elicited by their effect. However, the main and still open challenge is to find clues for discovering clear linkages between aldehyde-induced modifications of specific proteins and the development of various diseases.

## 1. Introduction

Aldehydes represent electrophilic carbonyl compounds with a hydrogen atom substituent on the carbonyl carbon [1]. The exposure of living cells to aldehydes may occur easily as the compounds are produced in metabolism or occur as natural dietary constituents, contaminants, drugs and pollutants. The major sources of endogenous aldehydes are lipid oxidation, oxidative degradation of amino acids (e.g., via polyamines) and sugar metabolism [2]. These metabolic products belong to a complex group of compounds that can covalently modify or cross-link proteins, yielding advanced lipoxidation end products (ALEs) and advanced glycoxidation/glycation end products (AGEs). ALEs and AGEs are involved in the development and progression of different oxidation-based diseases such as atherosclerosis, chronic renal failure, neurological disorders and diabetes [2]. For example, methylglyoxal (pyruvaldehyde) is formed as a byproduct in glycolysis but can also arise from the metabolism of threonine, fatty acids or keto compounds such as acetoacetate and acetone [3]. Its increased formation occurs under hyperglycemia, which is associated with, e.g., diabetes, liver and kidney diseases. Exogenous sources include food processing (cooking, fermentation) and storage as well as combustion processes. In proteins, methylglyoxal primarily reacts with Arg, Lys, Cys and Trp residues [2].

Reactive short-chain aldehydes, which arise as secondary products after the peroxidation of polyunsaturated fatty acids in lipids under oxidative stress conditions, can be classified into several groups [4]: alkanals, alkenals, substituted alkenals and alkanedials (dialdehydes, e.g., malondialdehyde). 2-alkenals (e.g., acrolein, crotonaldehyde) contain an α-β double bond in addition to the carbonyl function; substituted alkenals contain an extra hydroxyl or oxo group (e.g., 4-hydroxy-2-nonenal, 4-oxo-2-nonenal). Acrolein is also an environmental pollutant released by smoking cigarettes, the combustion of fuels, the production of plastics and the prolonged heating of frying oils [2,5]. It has been shown to inhibit cell proliferation, enhance apoptosis and disrupt gene expression. There is an association with several inflammation-related diseases such as atherosclerosis and Alzheimer’s disease [6]. The α,β-unsaturated carbonyl species react covalently with Cys, His, Lys and Arg residues in proteins [5]. Simple Michael adducts can react with another acrolein molecule to form cyclic products [7,8]. These aldehydes can promote protein carbonylation. Thus, determining the presence of carbonyl groups in proteins has been widely used to measure the level of oxidative stress [9].

**Figure 1:**
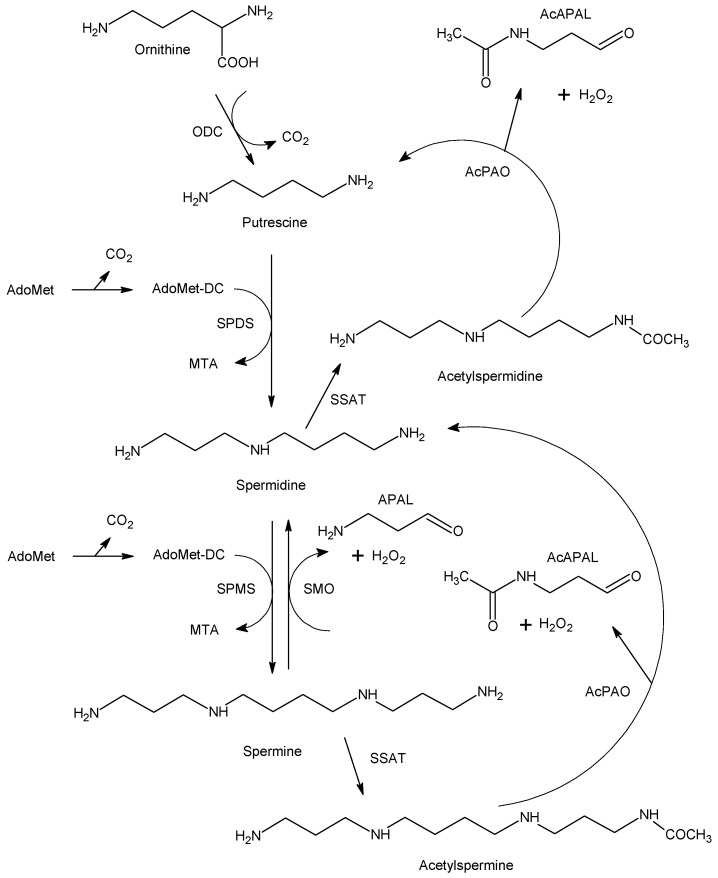
Polyamine metabolism in mammals. Both the biosynthesis and biodegradation of polyamines are shown. Abbreviations: AcAPAL, 3-acetamidopropanal; AcPAO, *N*^1^-acetylpolyamine oxidase; AdoMet, *S*-adenosylmethionine; AdoMet-DC, decarboxylated *S*-adenosylmethionine; APAL, 3-aminopropanal; MTA, 5′-methylthioadenosine; ODC, ornithine decarboxylase; SMO, spermine oxidase; SPDS, spermidine synthase; SPMS, spermine synthase; SSAT, spermidine/spermine *N*^1^-acetyltransferase 1. Note the formation of the aminoaldehydes APAL and AcAPAL in the oxidative reactions. This scheme was adapted from Casero et al. [11].

Polyamines have been recognized as important regulators involved in various growth and developmental processes (e.g., cell proliferation and differentiation, embryogenesis) and in responses to environmental stress [10,11,12,13]. Intracellular polyamine concentrations are maintained by a highly regulated metabolic network (at multiple levels, starting from gene transcription) consisting of biosynthetic and catabolic enzymes (Figure 1) as well as transport systems for both import and export. Putrescine (1,4-butanediamine; PUT), which is produced by ornithine decarboxylase (EC 4.1.1.17), is converted to spermidine (4-azaoctane-1,8-diamine; SPD) and spermine (4,9-diaza-1,12-dodecanediamine; SPM) by the corresponding synthases (EC 2.5.1.16 and 2.5.1.22, respectively). Decarboxylated *S*-adenosylmethionine provides the necessary aminopropyl groups [11]. There are three enzymes that facilitate polyamine catabolism in mammals, namely the inducible cytosolic spermidine/spermine *N*^1^-acetyltransferase 1 (SSAT, EC 2.3.1.57) and spermine oxidase (SMO, EC 1.5.3.16) and the constitutively expressed peroxisomal *N*^1^-acetylpolyamine oxidase (AcPAO, EC 1.5.3.13). The induction is based on various stimuli, including free polyamines, polyamine analogs, hormones, natural products and drugs. The above oxidases produce 3-aminopropanal (APAL) and 3-acetamidopropanal (AcAPAL), respectively, as well as hydrogen peroxide and the corresponding shortened polyamine. APAL can easily generate acrolein through the elimination of ammonia [14]. Elevated polyamine levels are needed for continued proliferation and tumor progression, especially in oncogene-driven cancers. Accordingly, antitumor polyamine analogs increase SSAT and SMO activities, which leads to a drop in cellular polyamines [15]. Copper-containing amine oxidases such as the intracellular kidney diamine oxidase (DAO; EC 1.4.3.22, formerly EC 1.4.3.6) and extracellular serum amine oxidase (SAO; EC 1.4.3.21, formerly EC 1.4.3.6 as well) can oxidize polyamines at their primary amino groups with a different efficiency. PUT belongs to the best substrates of DAO, which is in accordance with the name of the enzyme, but is only very weakly oxidized by SAO [16,17].

Protein modifications induced by the above-mentioned reactive aldehydes are analyzable using different analytical techniques, including mass spectrometry (MS), labeling with chemical probes (e.g., 2,4-dinitrophenylhydrazine) or immunochemistry with specific antibodies [4]. Immunochemical assays are best complemented by MS. The greatest advantage of MS is that it offers high sensitivity, high throughput of samples and provides structural information at the molecular level. Mass shifts caused by the formation of aldehyde adducts can be detected by protein or peptide MS. In addition, these modifications can be localized by tandem MS (MS/MS) at specific amino acid sites in the peptide sequences. On the other hand, several adducts may be overlooked due to their instability, especially Schiff adducts [4].

## 2. 3-Aminopropanal and Other Aminoaldehydes Produced in Polyamine Metabolism

**Figure 2:**
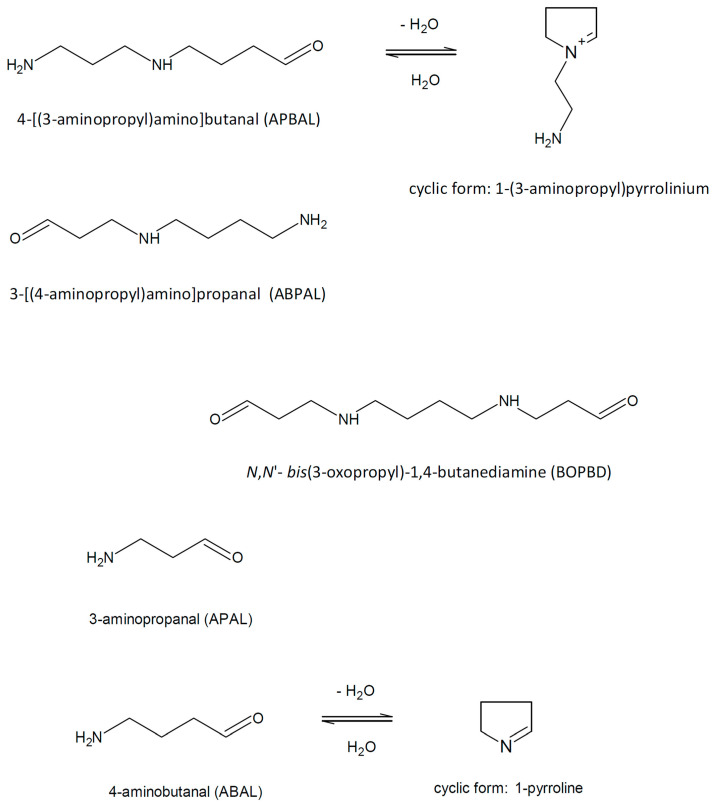
Polyamine-derived aminoaldehydes and their chemical structures.

Polyamine catabolism in living organisms is accompanied by the formation of several aminoaldehydes (Figure 2) such as *N*,*N*′-*bis*(3-oxopropyl)-1,4-butanediamine (BOPBD), *N*-(3-oxopropyl)-1,4-butanediamine, i.e., 3-[(4-aminobutyl)amino]propanal (ABPAL), 4-[(3-aminopropyl)amino]butanal (APBAL), 3-aminopropanal (APAL) and 4-aminobutanal (ABAL), as described further in the text.

BOPBD and ABPAL are produced by ruminant copper-containing SAOs when SPM and SPD are oxidized, respectively. The chemical nature of the aminoaldehydes was discovered by experiments with the bovine enzyme (BSAO) and clearly demonstrated that the oxidation occurred at the primary amino groups and resulted in the oxopropyl moieties of the products [18]. BOPBD and ABPAL were analyzed using paper chromatography after their reduction in the reaction mixture to more stable hydroxypropyl counterparts, and their chromatographic properties agreed with those of synthetic standards. Other chemicals properties of the reduced products, such as the melting points or infrared spectrum (measured for BOPBD), were also in accordance with the standards [18]. All of the above aminoaldehyde compounds were purified from BSAO reaction mixtures by means of an ion-exchange chromatography on SE-Sephadex. The purified aminoaldehydes (ca. 90% purity) were analyzed using thin layer chromatography (TLC) after their reduction to the corresponding hydroxypropyl derivatives by sodium borohydride and were assigned to the synthetic standards. Stability tests confirmed their spontaneous decomposition yielding acrolein and demonstrated the formation of larger condensation products [19].

High-performance liquid chromatography (HPLC) of rat tissue extracts with a post-column derivatization of amines by dansyl chloride and fluorescence detection demonstrated that SPD is oxidized by DAO at its terminal amino groups, yielding ABPAL or APBAL, which are converted via an aldehyde dehydrogenase (ALDH) reaction into the amino acids putreanine and isoputreanine, respectively [20]. The latter compound is formed by the hydrolysis of *N*-(3-aminopropyl)pyrrolidin-2-one, which was detected as an intermediate. APAL is produced by the oxidative deamination of 1,3-propanediamine (DAP), as has been shown using the ion-exchange chromatography of the reaction mixture containing this diamine and DAO from porcine kidney [21]. Similarly, APAL was purified from the reaction mixture of this enzyme through chromatographic steps and was obtained as a crystalline 2,4-dinitrophenylhydrazone. This colored product was analyzed using TLC and found to be identical to a derivative produced from enzymatically oxidized (by an alcohol dehydrogenase) 3-aminopropanol and also to a derivative isolated from blood serum after treatment with As_2_O_3_ and 2,4-dinitrophenylhydrazine (DNPH) [22]. DAP is released in the reaction of DAP-forming PAOs, which occur in plants and bacteria (see below). Additionally, it is supposed to be produced in vertebrates by the oxidative cleavage of isoputreanine, which is formed by APBAL oxidation, at the secondary amino group [23]. To the best of our knowledge, however, such a reaction has not yet been characterized. In bacteria, the formation of DAP is possible via 2-oxoglutarate 4-aminotransferase and L-2,4-diaminobutanoate dehydrogenase (from oxaloacetate, the C4 pathway) or via spermidine dehydrogenase (from L-glutamate, the C5 pathway). A transgenic *Escherichia coli* strain expressing the *dat* and *ddc* genes from *Acinetobacter baumannii* to introduce the C4 pathway and carrying other genetic optimizations was able to produce DAP at a level of 13 g/L [24].

A report based on ^1^H-NMR plus liquid chromatography data and describing the formation of APAL by BSAO in a successive conversion of SPM into SPD and SPD into PUT [21] was later challenged by pointing to sources of possible experimental artifacts. Lee and Sayre performed a reaffirmation of the chemical structure of the aminoaldehyde products of a BSAO reaction with SPM and SPD [25]. Uncertain action of BSAO on the secondary amino groups of the substrates was ruled out as homospermidine conversion by the enzyme yielded cyclic 1-(4-aminobutyl)pyrrolinium (no β-elimination of acrolein is possible in this case because of the non-existing oxopropyl moieties in the aminoaldehyde), indicating that the oxidative deamination occurred at the primary amino group. They also demonstrated by ^1^H-NMR that acrolein, which is released by the spontaneous decomposition of aminoaldehyde products generated from SPM and SPD (i.e., BOPBD and ABPAL, respectively), can react with ammonia and produce APAL, which results in the biasing of the interpretation of the chromatographic and NMR data acquired with reaction mixtures. Another NMR-based study of substrate oxidation by BSAO confirmed that the enzyme oxidized SPD and homospermidine at the primary amino groups [26]. Clear ^1^H-NMR signals of hydrated ABPAL were obtained. The authors further demonstrated that the presence of the *N*-aminopropyl group in BSAO substrates is reflected in their cytotoxic effect on human endothelial cells [26].

**Figure 3:**
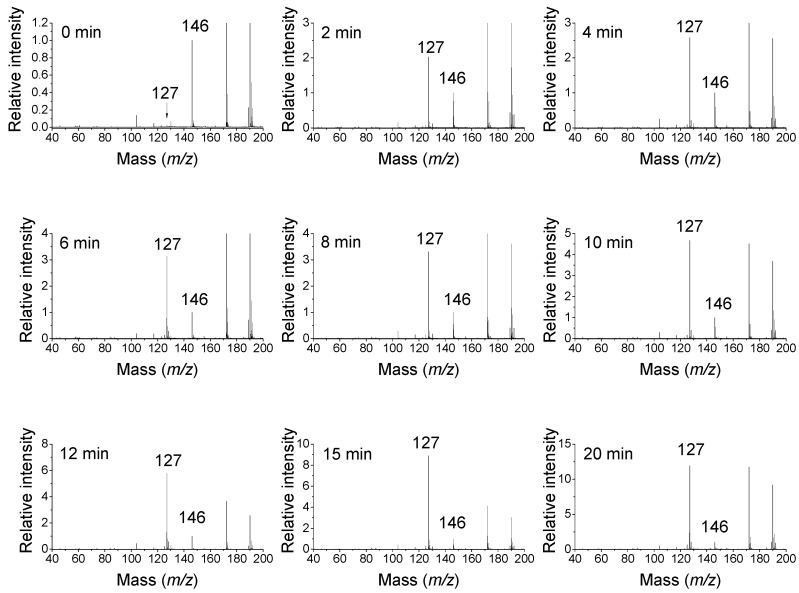
Spermidine oxidation by pea seedling DAO. SPD (1 mM) was oxidized in 50 mM of NH_4_HCO_3_ (adjusted to pH 7.0 by acetic acid) by adding 5 µL of the enzyme with a specific activity of 415 nkat/mg (PUT as a substrate) and protein content of 2.8 mg/mL. The reaction mixture was incubated at 37 °C and monitored over time by analyzing the withdrawn aliquots in a Microflex LRF20 MALDI-TOF mass spectrometer (Bruker Daltonik). The mass spectra are shown for a period of 0–20 min as indicated. α-Cyano-4-hydroxycinnamic acid was used as a matrix in the presence of cetyltrimethylammonium bromide to suppress matrix-derived signals, such as those with *m*/*z* 172 and 190 [31]. The SPD signal (*m*/*z* 146, [M + H]^+^) decreases, which is accompanied by an increasing 1-(3-aminopropyl)pyrrolinium product signal (*m*/*z* 127, M^+^). This is the authors’ own work.

ABAL emerges from the reaction of PUT oxidation by DAOs [27]. However, the free aldehyde form of ABAL is difficult to detect and quantify as a metabolite due to the inherent reactivity [28]. SPD is a good substrate of plant DAOs [16]. The expected product aminoaldehyde is APBAL in this case, which is in contrast to the above-mentioned reaction of BSAO producing the isomeric and oxopropyl group-containing ABPAL. ABAL and APBAL are known to cyclize spontaneously to 1-pyrroline and its *N*-aminopropyl derivative, i.e., 1-(3-aminopropyl)pyrrolinium, respectively, in the reaction mixtures of DAO or PAO [25,29]. As a proof, MALDI-TOF (matrix-assisted laser desorption ionization time-of-flight) mass spectrometry demonstrates that SPD (*m*/*z* 146) is oxidized by pea seedling DAO to a product with *m*/*z* 127 (Figure 3), suggesting a water loss and cyclization as the observed mass is consistent with the structure of the cationic 1-(3-aminopropyl)pyrrolinium. Aqueous solutions of ABAL were characterized by NMR spectroscopy in the pH range of 1 and 13 to demonstrate the existence of two neutral species (1-pyrroline, pyrroline trimer) and four protonated species (1-pyrrolinium, free aldehyde, hemiaminal and hydrate). The pyrroline (predominant) and its trimer were present at basic pH values, whereas the open-chain forms, pyrrolinium and hemiaminal, appeared at the pH range of 1–6. Interestingly, 4-butylaminobutyraldehyde exclusively existed as *N*-butylpyrrolinium ion at acidic pH [28]. Lentil seedling DAO generates BOPBD and APBAL by the oxidative deamination of SPM and SPD, respectively. In the presence of high SPM amounts, BOPBD was shown to react with the primary amino groups of other SPM molecules, yielding a yellow-orange compound. Its analysis using MS and two-dimensional NMR experiments allowed for the identification of a pyrimidine-based cyclic structure with two side chains [30].

The maize enzyme is a representative of the class of DAP-forming PAOs (EC 1.5.3.14) from the monocotyledonous plants. These enzymes oxidize SPM to APBAL, hydrogen peroxide and DAP. SPD is oxidized to ABAL, H_2_O_2_ and DAP [32]. Because these products cannot be converted directly to other polyamines, the DAP-forming PAOs are considered to be involved in the terminal catabolism of polyamines. DAP formation was demonstrated by TLC of the reaction mixture components with a fluorescence detection upon dansylation [32]. In mammals, the cytosolic SMO (EC 1.5.3.16) is a highly inducible flavoprotein enzyme that participates in polyamine homeostasis. It exclusively oxidizes SPM and not SPD or *N*^1^-acetyl-SPM (the specificity is charge-based) and produces SPD, hydrogen peroxide and APAL as reactions products [33]. Non-specific PAOs (EC 1.5.3.17) oxidize SPM and SPD but may also accept acetylated polyamines such as *N*^1^-acetyl-SPM or *N*^1^-acetyl-SPD as substrates. Additionally, their reaction resembles that of mammalian APAL-producing PAOs. Examples have been found to occur in, e.g., *Arabidopsis* [34,35]. *N*^1^-acetylated SPM and SPD are preferentially oxidized by mammalian peroxisomal AcPAOs (EC 1.5.3.13). *N*^1^-acetyl-SPM and *N*^1^-acetyl-SPD are converted to SPD and PUT, respectively, 3-acetamidopropanal (AcAPAL) and H_2_O_2_ [36,37]. This process is known as polyamine back-conversion because of the formation of deacetylated polyamines. Maize PAO (EC 1.5.3.14) oxidizes *N*^1^-acetyl-SPM to 4-[(3-acetamidopropyl)amino]butanal and *N*^8^-acetyl-SPD to AcAPAL. The other products are DAP and H_2_O_2_ [32].

## 3. Cytotoxicity of Polyamine-Derived Aminoaldehydes

Polyamine-derived aminoaldehydes were purified on a cation exchanger and used in experiments, demonstrating their toxicity to the malarial parasite *Plasmodium falciparum* that was cultured in erythrocytes and treated at the young and mature developmental stages [38]. A strong lethal effect of the aminoaldehydes was observed at >100 µM concentrations after incubation for 105 min by inhibiting the incorporation of [^3^H]hypoxanthine, and it was more pronounced in the mature parasite [38]. Bovine serum is frequently used as a medium supplement for in vitro cell cultures. When SPD (30 μM) was exogenously added to cultured mouse cells in the presence of bovine fetal or calf serum, the contained BSAO activity caused considerable cytotoxic effects because of the generation of the products aminoaldehyde and hydrogen peroxide. This SPD-related toxicity was found to be dose-dependent and was inhibited by 1 mM of aminoguanidine as an inhibitor of BSAO [39]. The cytotoxic effects of the SPM oxidation products were analyzed in a study with mouse FM3A cells in the presence of bovine calf serum [40]. Exogenously added SPM (15 and 30 µM) as well as acrolein (7.5 and 15 µM), which is expected to be formed by the spontaneous decomposition of SPM-derived aminodialdehyde, largely inhibited FM3A cells growth. A similar effect was found for APAL at concentrations of 25–50 µM and SPD at concentrations of 50–100 µM. Other aldehydes, such as formaldehyde, acetaldehyde or propionaldehyde, were required at much higher concentrations (10^−4^–10^−3^ M), and this was similar for hydrogen peroxide. Aminoguanidine and yeast ALDH degrading aldehydes prevented these inhibitions, suggesting their connection with the reactive aldehyde products of polyamine metabolism (catalase had no or minimal effect) [40]. The formation of acrolein in the reaction mixture of BSAO or during the spontaneous decomposition of APAL was demonstrated by a previously developed fluorescence-based assay method with *m*-aminophenol [41].

A mouse lymphocytic leukemia cell line was used to study the cytotoxicity of the aminodialdehyde BOPBD, which was produced by BSAO reacting with SPM [42]. SPM exerted cytotoxicity at a concentration of 25 µM, and complete cell death was observed within 12 h. The cytotoxicity was prevented by aminoguanidine. Catalase delayed but did not prevent cell death by hydrogen peroxide scavenging; thus, its primary determinant was likely the aminoaldehyde generation. Reduced glutathione (GSH) was found to be the major target of both SPM oxidation products. The attributes of the observed cell death process (e.g., a lack of caspase activation involvement) as well as cell morphology indicated necrosis rather than apoptosis but with damage to mitochondria and phosphatidyl serine redistribution at the plasma membrane [42]. Exogenously added SPD has been shown to promote longevity by inducing autophagy in a series of in vitro studies with yeast, *Drosophila* and murine models [43]. Experiments with a lung adenocarcinoma cell line and the autophagy marker protein LC3B (human microtubule associated protein 1 light chain 3), which binds to autophagosomal membranes in its form LC3B-II, showed that the autophagy induction by SPD and SPM only occurred in the presence of active BSAO from supplemental bovine serum. Hence, the frequently observed autophagic responses to exogenous polyamines seem to be artifacts [44].

The levels of PUT, SPD, SPM and acrolein were analyzed in the plasma of patients with renal failure. PUT was approximately increased twice compared with normal subjects, whereas SPD and particularly SPM were decreased, as determined by an HPLC analysis. This correlated with the observed increase in PAO activity [45]. Both free and protein-bound acrolein were analyzed by HPLC and by using specific antibodies, respectively. Interestingly, their levels were well correlated with the renal failure severity. The increased level of the free aldehyde was slightly above 1 µM compared with 0.5 µM in normal subjects; the conjugated acrolein reached about 170 µM, which is a five-fold increase [45]. Polyamine oxidation, represented by the activity levels of AcPAO and SMO as well as acrolein concentration, reflected in the formation of *N*^ε^-(3-formyl-3,4-dehydropiperidino)lysine (i.e., FDP-Lys adducts of proteins), was found to be elevated in the plasma of stroke patients and was correlated with the size of stroke. Hence, these levels were suggested as early diagnostic markers of the disease [46]. Both enzymes were assayed by HPLC, and the protein-conjugated acrolein was determined using specific antibodies in ELISA (enzyme-linked immunosorbent assay) tests. An increase in AcPAO occurred first, followed by increased SMO and then FDP-Lys [46]. Cigarette smoking has been long known to suppress immune responses in the lungs. Smokers are also more susceptible to respiratory infections [47]. The vapor phases from cigarette smoke extracts were found to inhibit the production of several proinflammatory cytokines in macrophages and T-cells. Both acrolein and crotonaldehyde, identified in the vapor phase by gas chromatography coupled with mass spectrometry (GC-MS) and quantified in the whole extract by reversed-phase HPLC after derivatization with 2,4-dinitrophenylhydrazine, exerted 50% inhibitory concentrations (IC_50_) at micromolar levels. The inhibition by these α,β-unsaturated aldehydes was completely abrogated by *N*-acetylcysteine. The inhibition of cytokine production was associated with affecting the expression of related genes, as demonstrated by an mRNA-level analysis [47]. In a follow-up study, direct covalent modifications of the transcription factor NF-κB1 (p50) by acrolein at Cys61 and Arg307 (among other modified residues) were demonstrated by MS/MS, which structurally elucidated the observed blocking of its DNA-binding abilities after the reaction, as analyzed by an electrophoretic mobility shift assay [48].

**Figure 4:**
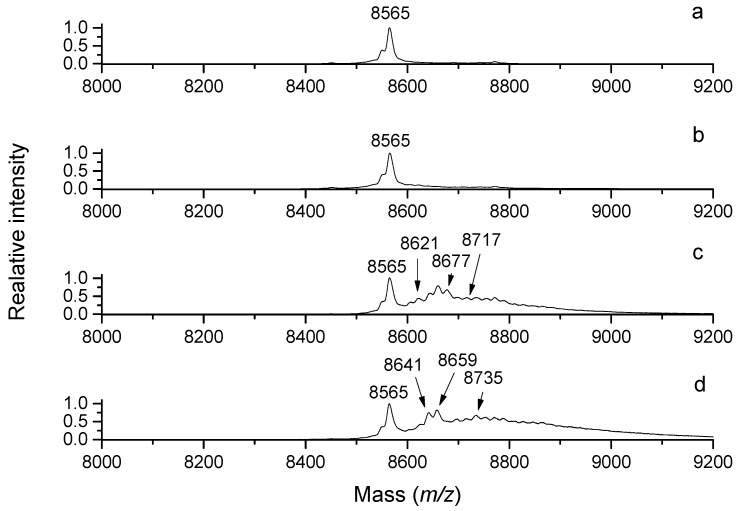
Chemical modifications of ubiquitin by 3-aminopropanal and acrolein. Bovine ubiquitin (100 µM) was incubated with 5 mM of APAL or acrolein in 50 mM of NH_4_HCO_3_ at 37 °C for up to 48 h. The MALDI-TOF mass spectra of the reaction mixtures were acquired on a Microflex LRF20 MALDI-TOF mass spectrometer (Bruker Daltonik) using a binary matrix composed of ferulic and sinapinic acids (5 and 15 mg/mL, respectively, in acetonitrile:2.5% trifluoroacetic acid; 7:3, *v*/*v*). From the top: (**a**) unmodified ubiquitin (*m*/*z* 8565, [M + H]^+^); (**b**) ubiquitin reacted with APAL for 48 h; (**c**) ubiquitin reacted with acrolein for 2 h; (**d**) ubiquitin reacted with acrolein for 48 h. Note the presence of multiple adducts, including, e.g., *m*/*z* 8603 (8565+38), 8621 (8565+56), 8641 (8565+76), 8659 (8565+94), 8677 (8565+56+56), 8697 (8565+56+76), 8717 (8565+76+76), 8735 (8565+76+94) and others in the reaction mixture containing acrolein. Their presence in the reaction mixture containing APAL is documented as well, but it is much less obvious due to lower abundance. This is the authors’ own work.

Serum albumin, which is an abundant protein in blood serum (and also in lung lining fluid), has multiple physiological functions including the binding and transportation of molecules and ions, regulation of colloid osmotic pressure and maintenance of vascular pressure [49]. The carbonylation of bovine serum albumin (BSA) upon its reaction with acrolein was studied using a colorimetric assay with DNPH, sodium dodecyl sulfate polyacrylamide gel electrophoresis (SDS-PAGE), Western blotting with anti-dinitrophenylhydrazone-adduct antibodies and nanoflow liquid chromatography coupled with electrospray ionization tandem mass spectrometry (nanoLC-ESI-MS/MS). Furthermore, this study was interested in the influence of acrolein on gene expression and its cytotoxicity towards cultured lung epithelial cells in the presence or absence of BSA as an aldehyde scavenger [50]. SDS-PAGE followed by Western blotting demonstrated the formation of carbonylated and cross-linked BSA forms with increased molecular masses after the reaction with acrolein. It was shown that the carbonylation of BSA by acrolein decreased the increased the transcription of two selected genes (a transcription factor, heme oxygenase 1), which was induced by acrolein itself. An increased in vivo BSA carbonylation was demonstrated in the bronchoalveolar lavage of rats exposed to acrolein. The in vivo adduction site in BSA was then found at Cys34 using nanoLC-ESI-MS/MS [50]. Similarly, multiple modifications of bovine ubiquitin by acrolein are detectable using MALDI-TOF MS (Figure 4). Acrolein has been shown to inactivate various enzymes, such as protein tyrosine phosphatases (EC 3.1.3.48) [51]. These enzymes dephosphorylate phosphotyrosine residues in proteins via a phosphocysteine intermediate and participate in cell signaling. Kinetic measurements with a recombinant enzyme showed that the inactivation occurred in a time-dependent manner. MS/MS analyses of the reacted enzyme digested to peptides using both a MALDI-TOF/TOF and Q-TOF (quadrupole time-of-flight) instrument allowed for the recognition of active-site Cys215 as the site of modification [51]. Alzheimer’s disease has been associated with oxidative stress in the brain, which is accompanied by an increase in lipid peroxidation, protein and DNA oxidation together with a decrease in energy utilization [52]. Interestingly, acrolein inhibits the activity of the mitochondrial enzymes pyruvate dehydrogenase (EC 1.2.4.1) and 2-oxoglutarate dehydrogenase (EC 1.2.4.2), which could potentially contribute to the neurodegenerative displays of the disease. This inhibition was elucidated by the covalent modification of lipoic acid, which is a cofactor of the enzymes [53].

## 4. Cytotoxicity of 3-Aminopropanal

Cerebral ischemia is caused by insufficient blood flow in the brain tissue, which leads to hypoxia and subsequently cell death. The area surrounding an ischemic event is called a penumbra [54]. The polyamines SPD and SPM are abundant brain metabolites, and they are implicated in the pathogenesis of the disease as their increased oxidation by upregulated PAO is accompanied by the production of APAL. The p*K*_a_ value of APAL, which is approximately 9.3, suggests its lysosomotropic properties. A high intralysosomal concentration of the aminoaldehyde upon its accumulation is supposed to lead to reactions with thiol groups in proteins and subsequent damage to lysosomal integrity [55,56]. Ivanova et al. studied APAL as a cytotoxic mediator in ischemic rats [57]. They developed an HPLC method to quantify the compound in tissue samples as a 2,4-dinitrophenylhydrazone adduct. The APAL levels were found to be significantly elevated 2 h after the onset of ischemia, and the increase continued with cell death development and spreading around the ischemic core. Unlike glial cells, where apoptosis was activated via caspase 1, APAL did not induce apoptosis in neuronal cells but primarily caused necrosis. Adding PAO inhibitors effectively prevented ischemic cell damage [57]. In another study, a human neuroblastoma cell line (SH-SY5Y), which resembles neurons, was treated by 10–50 µM of APAL to evaluate the induced cell death process by cytological approaches such as fluorescence microscopy or flow cytometry. Mixed signs of apoptosis and necrosis were already seen at a concentration of 20 µM [56]. Ammonium chloride (30 mM) was found to protect against APAL toxicity by preventing its lysosomal accumulation. Another protective effect has been documented for α-tocopherol, which is a radical scavenger. Its application delayed the observed production of reactive oxygen species but did not interfere with the early phase of lysosomal rupture leading to the release of lysosomal hydrolases [56].

A keyhole limpet hemocyanin was reacted with APAL in the presence of sodium borohydride and used to raise antibodies using rabbit immunization. These antibodies were then applied to detect and semi-quantify (by dot blotting using APAL-reacted BSA as a standard) the level of APAL-modified proteins in biological samples, such as in the cerebrospinal fluid of ischemic patients [58]. Patients with a good neurological prognosis had significantly lower levels of such protein modifications compared with those with the most severe forms of disease. The authors additionally screened a library of sulfhydryl compounds and found that *N*-(2-mercaptopropionyl)glycine (*N*-2-MPG) efficiently neutralized APAL in glial and neuronal cell cultures by forming a thioacetal product. *N*-2-MPG was also found using immunohistochemistry to significantly reduce the cytotoxicity of APAL in vivo in a rat ischemic model with middle cerebral artery occlusion [58]. Phenelzine is another example of a possible aldehyde sequestration reagent, which is efficient against both APAL and acrolein cytotoxicity. In this case, a hydrazone is produced by the reaction. Phenelzine avidly neutralizes APAL in retinal ganglion cells, even when administered with a delay of several hours [59]. *N*-2-MPG is an approved drug (tiopronin) for the treatment of cystinuria. It was applied as a study drug to treat aneurysmal subarachnoid hemorrhage [60]. No adverse effects were registered in phase I clinical trials. All enrolled patients were administered the compound within 96 h of the hemorrhage onset for a short period of 14 days. The patients were otherwise subjected to surgical or endovascular repair of their aneurysm and a standard therapy for cerebral vasospasm [60]. The efficacy of tiopronin to neutralize APAL in cerebrospinal fluid was evaluated in a subsequent phase II study utilizing LC-ESI-MS/MS to quantify the compound [61]. Delayed cerebral ischemia is a significant cause of morbidity and mortality among patients who survive the initial aneurysmal rupture. Unfortunately, no significant difference was found when comparing APAL levels and other measured clinical data for the patients who received tiopronin and those who received placebo.

The molecular mechanism of APAL cytotoxicity, which is related to the apoptosis of neurons and glial cells as a consequence of degenerative or traumatic injuries to the central nervous system, was studied using histochemistry and immunohistochemistry to demonstrate that the compound primarily targets lysosomes [55]. Human astrocytoma cells were used as a model and were subjected to microscopy after the treatment with 75 µM of APAL, resulting in apoptosis. Specific antibodies allowed for the localization of high levels of APAL-modified proteins in lysosomes. The occurrence of lysosomal ruptures was demonstrated by a fluorescence staining with acridine orange, increased immunoreactivity of the lysosomal cathepsin B inside the whole cell, caspase activation and typical morphological signs of apoptosis. These effects were inhibited by the simultaneous additions of NH_4_^+^ and *N*-2-MPG, which yielded a reduced accumulation of APAL in lysosomes because of their alkalization and trapping of the aminoaldehyde in a non-toxic thioacetal adduct, respectively [55]. It has been postulated that the aldehyde functional group of APAL is required for its cytotoxicity, whereas the amino group is required for the lysosomal uptake.

Microscopic experiments with marker dyes and lysosome-rich murine macrophage cells demonstrated the lysosomotropic properties of APAL [62]. Treating cells with 100 µM of APAL resulted in a time-dependent increase in apoptosis. The induced lysosomal rupture led to a mitochondria-associated and enhanced production of reactive oxygen species, which was likely due to processes involving the release of cysteine-containing cathepsins, as their specific inhibition largely reduced the observed APAL-induced effects. Early lysosomal ruptures were detectable already after a short exposure to 100 µM of APAL for only 20 min, and apoptotic cells and caspase activation were observed after 3 h of treatment, indicating that apoptosis was not induced immediately. Necrosis was the predominant cell death form when a higher concentration of 200 µM of APAL was applied [62]. Several pieces of experimental evidence were obtained that indicated that the late endosomal/lysosomal membrane-associated protein Niemann–Pick C1 (NPC1) was involved in regulating the accumulation of amines in lysosomes [63]. Microscopic data for human fibroblasts demonstrated that NPC1 mediated the vacuolization of lysosomes induced by lysosomotropic amines. Moreover, these amines facilitated the formation of late endosomal/lysosomal hybrid organelles in the presence of NPC1 (monitored by fluorescence resonance energy transfer after lysosomal endocytosis of a fluorescently labeled and biotinylated dextran and the streptavidin-conjugated labeled latex beads localized in endosomes), and the subsequent lysosomal cargo secretion was stimulated. NPC1-depleted fibroblasts were found to be more susceptible to the toxic effects of APAL manifested by damage to the lysosomal membrane [63]. A rat retinal ganglion cell line was used as a model to compare the cytotoxicity of APAL and AcAPAL [64]. The cytotoxic effect was assayed by measuring the activity of lactate dehydrogenase (EC 1.1.1.27). The enzyme is cytosolic and is released into the cell culture medium upon damage to the plasma membrane. While APAL showed a strong effect at a concentration of 200 µM (although it was much less pronounced than those of acrolein or hydrogen peroxide at the same concentration), AcAPAL displayed no cytotoxicity up to a concentration of 1 mM.

## 5. Analysis of Protein Modifications Induced by Reactive Aldehydes

**Figure 5:**
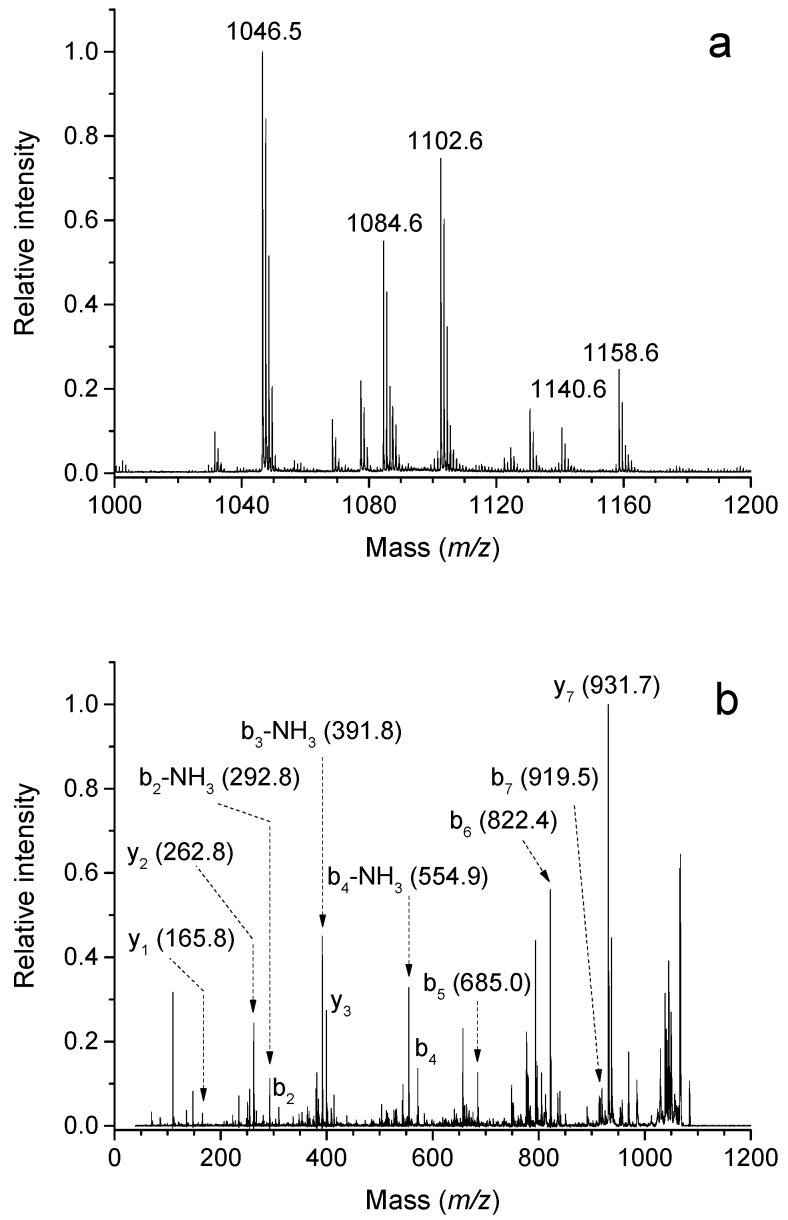
MALDI mass spectrometry of acrolein-modified angiotensin II. Human angiotensin II (100 μM) was reacted with 5 mM of acrolein in 50 mM of NH_4_HCO_3_ at 37 °C for 2 h. Then, the reaction mixture was analyzed using an ultrafleXtreme MALDI-TOF/TOF mass spectrometer (Bruker Daltonik) using α-cyano-4-hydroxycinnamic acid as a matrix. Panel (**a**) shows a mass spectrum with signals of angiotensin II (*m*/*z* 1046) and its adducts with acrolein (*m*/*z* 1084, 1102, 1140, 1158). Panel (**b**) shows a fragmentation pattern of the modified peptide *m*/*z* 1084 acquired after laser-induced dissociation (a LIFT-TOF/TOF spectrum), which demonstrates the presence of a Schiff base modification at the N-terminus. This is the authors’ own work.

To the best of our knowledge, protein modifications induced by APAL have not yet been studied at the molecular level except for the above-mentioned detections with antibodies [55,58]. Cys, Lys and His residues in particular can be expected to be sensitive as it has been documented for the reactions of other reactive aldehydes such as methylglyoxal, acrolein and crotonaldehyde [7,65,66]. Of course, when the expected elimination of ammonia from APAL produces acrolein [14], the effect of acrolein must then be taken into consideration. Parameters such as relative softness and electrophilicity, which are based on the theory of hard and soft acids and bases and are derived from quantum chemical calculations, were used to evaluate different aldehydes for their reactivity toward protein nucleophiles [1]. Electrophiles preferentially and more rapidly form covalent adducts with nucleophiles of comparative softness or hardness. Hard electrophilic aldehydes (e.g., formaldehyde and alkanals) are relatively non-polarizable (their low electron density is localized on the carbonyl carbon atom), while soft electrophilic aldehydes (e.g., acrolein) are more polarizable (they contain multiple sites with low electron density). Cys residues are soft nucleophiles, whereas Lys and His are relatively harder nucleophiles. Different subclasses of aldehydes from the same group (e.g., various α,β-unsaturated aldehydes) can provide additive toxic effects [1]. MS/MS measurements are well suited for the detection and identification of protein modifications using reactive aldehydes (Figure 5). The most typical methods are bottom-up strategies with a protein digestion step. Due to their low abundance, it is usually necessary to fractionate modified peptides by selective enrichment and use separation methods to improve detection sensitivity. The hydride reduction of the aldehyde moiety in the Michael-type adducts is often used to stabilize the modification before its loss, making it amenable to collision-induced dissociation MS/MS analysis [67].

To identify the mechanism of methylglyoxal reaction with proteins, the compound was reacted at neutral pH with *N*^α^-acetylamino acids and BSA [68]. The reactions were studied using ^1^H-NMR, UV spectrophotometry, and [^14^C]-methylglyoxal and ultrafiltration/precipitation to detect modifications in BSA. *N*^α^-acetyl-L-Arg, *N*^α^-acetyl-L-Cys and *N*^α^-acetyl-L-Lys were reacted to form imidazolone (irreversible modification), hemithioacetal and bis-glycosylamine derivatives, respectively. The imidazolone was isolated using preparative HPLC and characterized using ^1^H-NMR. With respect to BSA, 55% of the original methylglyoxal was irreversibly bound to the protein after 4 days [68].

2-Alkenals, including acrolein and crotonaldehyde, are unsaturated and highly reactive aldehydes containing two electrophilic centers at the carbon atoms 1 and 3, which can bind to nucleophilic residues in proteins [7,66]. BSA was subjected to acrolein-induced modifications. The protein was found to react at Lys and His residues, and the reaction introduced carbonyl groups that were detectable by a colorimetric assay with DNPH. Accordingly, acrolein was reacted with *N*^α^-acetyl-L-Lys and *N*^α^-acetyl-L-His to analyze the chemical structures of the resulting modification products [7]. The product compounds were isolated using reversed-phase high-performance liquid chromatography (RP-HPLC), and their ^1^H- and ^13^C-NMR spectra were assigned to *N*^α^-acetyl-*N*^ε^-(3-formyl-3,4-dehydropiperidino)lysine, i.e., *N*^α^-acetyl-FDP-Lys and a 1-(3-oxopropyl) derivative of *N*^α^-acetylhistidine, respectively. Monoclonal antibodies were raised against acrolein-modified hemocyanin. These antibodies were found to recognize FDP-Lys as an epitope in acrolein-reacted proteins and were then applied for immunodetection to pathological samples such as atherosclerotic tissues. Polyunsaturated fatty acids oxidized during oxidative stress and initiated experimentally by metal ions have been shown to be potential sources of acrolein [7]. Interestingly, FDP-Lys in acrolein-modified proteins readily reacts with thiols such as cysteine, glutathione and even cysteine residues in other proteins, including those representing the catalytic sites in enzymes (e.g., glyceraldehyde phosphate dehydrogenase), to yield cross-links and aggregates. The reaction mechanism involves a Michael-type addition of the thiol group to the double bond of FDP-Lys, and it is accompanied by a shift in the UV absorption maximum [69]. Crotonaldehyde adducts were studied using NMR spectroscopy after their isolation by HPLC, with *N*^α^-hippuryl-L-Lys and *N*^α^-acetyl-L-Lys being used as model compounds. β-Substituted butanal derivatives (Michael adducts) were confirmed as reaction products. The hippuryl lysine additionally reacted with one more crotonaldehyde to form the corresponding dimethyl-FDP moiety. The free aldehyde groups in the adducts were analyzable by HPLC as 2,4-dinitrophenylhydrazones [66]. BSA modifications were analyzed by amino acid analysis and immunohistochemistry using a specific monoclonal antibody raised against crotonaldehyde-treated hemocyanin. The antibody recognized crotonaldehyde–lysine adducts (as well as adducts with 2-pentenal and 2-hexenal), including an additional Schiff base-derived 5-ethyl-2-methylpyridinium (EMP)-Lys. The immunoreactivity of the antibody in an in vivo study in mice under oxidative stress demonstrated that substantial amounts of 2-alkenals could be formed during the peroxidation of polyunsaturated fatty acids [66]. Many transcription factors utilize cysteine residues as redox switches within redox-dependent regulation mechanisms. Modifications of these cysteines by acrolein then interfere with the regulatory mechanisms of gene expression [5].

**Figure 6:**
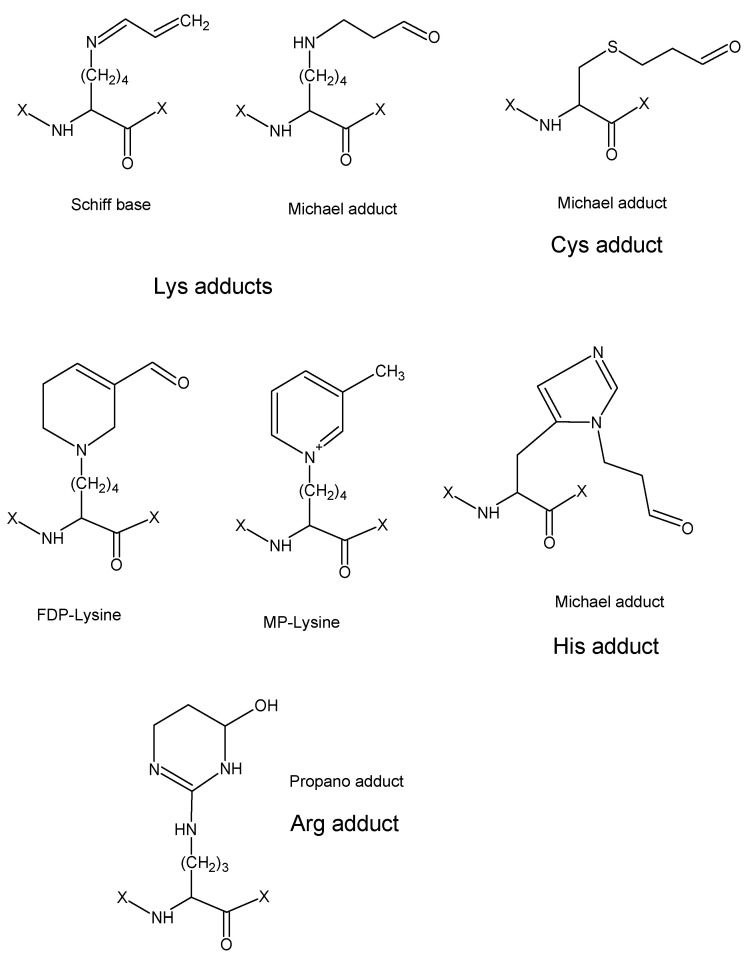
An overview of common protein adducts with acrolein. This scheme was adapted from the literature [5,8,70].

A similar pyridinium-containing lysine derivative generated from the binding of two acrolein molecules was identified as *N*^ε^-3-methylpyridinium (MP)-Lys using NMR spectroscopy. The formation of 3-methyl pyridinium moieties in the B-chain of insulin reacted with acrolein was demonstrated to occur at Lys29 and the N-terminal amino group (Phe) using liquid chromatography coupled to electrospray tandem mass spectrometry, i.e., LC-ESI-MS/MS [8]. This approach has repeatedly been shown to be powerful for both the identification and quantification of acrolein adducts in proteins [70]. Four types of such adducts with amino acids were reported based on MS-based data (Figure 6): (1) a Schiff base of Lys (aldimine), (2) Michael adducts of Cys, His and Lys, (3) FDP-Lys, and (4) MP-Lys [8,71,72]. The observed mass differences were + 38, 56, 94 and 76 Da, respectively. The unstable Schiff base of Lys as well as the Michael adducts and FDP-Lys could be reduced by sodium borohydride, increasing the mass differences to 40, 58, and 96 Da, respectively [70]. Unmodified peptides in trypsin digests generated upon protein modification by reactive aldehydes can be utilized as standards for relative quantification when a stable isotope-labeled internal standard is not available [70]. Functionalized aldehyde-reactive probes (e.g., with a hydrazine or hydroxylamine function and biotinylation) have been developed for the chemical derivatization and tagging of adduct aldehyde groups prior to MS/MS [67].

Methyl vinyl ketone (MVK) is a close structural analog of acrolein, which has an additional methyl group. The compound represents an efficient Michael acceptor for protein nucleophiles because of the α,β-unsaturation. ESI-MS/MS analyses of peptide modifications in the presence of MVK indicated that no cyclic adducts were formed in contrast to the effect of acrolein, which is in agreement with the expected steric hindrance for cyclization imposed by the α-methyl group [73]. MVK still caused the formation of mono- and bis-Michael adducts. Because MVK and acrolein displayed a similar cytotoxicity in hepatocytes and protein carbonylating efficiency, it seems that the formation of cyclic adducts is not a prerequisite for their acute toxicity [73]. Another short-chain aldehyde, pentanal, was shown to be much less reactive in comparison with acrolein. The target model proteins were lysozyme and human serum albumin (HSA). Whereas only Schiff bases of Lys were found in pentanal-reacted lysozyme or HSA, the acrolein binding (Michael adducts) occurred at Cys, Lys and His [71]. These modifications were investigated using a nanoLC-ESI-MS/MS analysis of peptides generated by the tryptic digestion of the reacted proteins, which allowed for the identification of the respective modification sites. The authors obtained several diagnostic fragment ions for the aldehyde adducts, which could be useful in targeted MS approaches [71]. ESI-MS/MS analysis of bovine insulin-derived peptides and various synthetic peptides containing Cys and Lys (or His) residues were reacted with acrolein and then analyzed for the formation of Michael adducts and their further transformation [74]. If the reacted Cys (M+56 Da, where M is the peptide molecular mass) appeared in proximity of the N-terminus (within seven residues), a Schiff base was formed by its reaction with the N-terminal amino group (M+38 Da). It has been postulated that such reactions may lead to intramolecular or intermolecular cross-linking reactions, inducing functional changes in proteins [74].

## 6. Degradation of Polyamine-Based Aldehydes

The degradation metabolism of aldehydes was comprehensively reviewed in a previous paper from 2005, including a detailed description of the enzymes and enzyme families that are involved [14]. Most aldehydes generated by lipid peroxidation, including 4-hydroxy-2-nonenal and potentially acrolein, react with GSH by Michael addition to form GSH conjugates, which are further metabolized. Aldehydes can also be reduced to alcohols by alcohol dehydrogenases (ADHs) or aldo/keto reductases (AKR). AKRs and aldehyde dehydrogenases, ALDHs, can not only convert the free aldehyde but also its Michael adduct with GSH [6]. Because acrolein is additionally formed by the elimination of ammonia from APAL plus SPD- and SPM-derived aminoaldehydes, their detoxification partially overlaps with that of acrolein. The reaction of acrolein with GSH yields *S*-(3-oxopropyl)-*N*-acetylcysteine after enzymatic cleavage of Glu and Gly from the primary conjugation product and *N*-acetylation. Its reduction by AKR provides *S*-(3-hydroxypropyl)-*N*-acetylcysteine (3-hydroxymercapturic acid, HPMA), the main acrolein metabolite found in urine, while the oxidation by ALDH yields *S*-carboxyethyl-*N*-acetylcysteine, i.e., carboxyethyl mercapturic acid [5]. It is a spontaneous reaction, but its efficiency is largely increased by the activity of glutathione-*S*-transferase. Acrolein can also be detoxified via nucleophilic ascorbylation, where ascorbate in its enolate form reacts as a nucleophile with the aldehyde in a Michael-type addition reaction [5].

Exogenously added catalase and ALDH protected mammalian cells from the cytotoxic effect of SPM oxidized in the presence of BSAO [75]. Catalase prolonged the 100% survival rate by 20 min, but then cell survival steadily declined, indicating that hydrogen peroxide is not the exclusive toxic compound. ALDH alone showed a partial protective effect in the later stages of BSAO reaction. The cytotoxicity induced by BSAO was completely inhibited only when both catalase and ALDH were present [75]. Mouse melanoma cells B16 were treated with SPM in the presence of BSAO, and the treatment was optionally performed with the additional presence of catalase and/or ALDH as well as caspase inhibitors [76]. Exogenous SPM (5–50 µM) already exerted cytotoxicity after incubation at 37 °C for 1 h. The type of cell death induced was investigated using fluorescence microscopy. A concentration of 50 μM of SPM induced apoptosis, whereas SPM concentrations above 100 μM predominantly caused necrosis. Catalase and ALDH reduced the observed toxicity, and the former additive was more effective, suggesting that H_2_O_2_ is the main cytotoxic agent followed by the aminoaldehyde oxidation product (i.e., BOPBD and the subsequently generated acrolein). The induction of apoptosis involved the activation of caspases 9 and 3, as demonstrated using specific substrates and inhibitors [76].

ALDHs are NAD^+^/NADP^+^-dependent enzymes, which oxidize their aldehyde substrates to the corresponding carboxylic acids. ALDHs constitute a superfamily, which consists of 24 distinct families across eukaryotic organisms [77,78]. This classification is based on the sequence identity level. Proteins sharing ≥40% identity belong to a particular family indicated by an Arabic numeral, whereas those sharing ≥60% identity are classified in the same subfamily with an additional letter code [79,80]. Interestingly, *ALDH* genes belonging to different *Pseudomonas* species (258 strains) could recently be sorted out into 42 families (!), from which 14 were found to be predominant [81]. The polyamine oxidation products APAL and ABAL are converted namely by representatives of the families ALDH9 (in microorganisms and animals) and ALDH10 (in plants), which are thus named aminoaldehyde dehydrogenases (AMADHs). AMADHs include betaine aldehyde dehydrogenases (EC 1.2.1.8), 4-aminobutyraldehyde dehydrogenases (EC 1.2.1.19), 4-trimethylaminobutyraldehyde dehydrogenases (EC 1.2.1.47) and 4-guanidinobutyraldehyde dehydrogenases (EC 1.2.1.54) in the Enzyme Commission nomenclature system (available at https://enzyme.expasy.org, accessed on 10 October 2023). Some bacterial enzymes from the ALDH25, ALDH26 and ALDH27 families also exhibit AMADH activity [78]. ALDHs are intracellular enzymes. For example, human ALDH9A1 is cytosolic [82]. The amino acid sequences of PsAMADH1 and PsAMADH2 from pea contain tripeptide peroxisome-targeting signals at the C-terminus [83]. Thus, in peroxisomes, AMADHs can directly degrade the aminoaldehydes produced by peroxisomal PAOs.

ALDHs are known as homotetramers or homodimers with a subunit mass of 50–60 kDa [84]. Each monomer contains a coenzyme-binding domain, a catalytic domain, and an oligomerization domain [85]. The active site, which appears at the interface of these domains opposite the coenzyme binding site, is accessible through a funnel-shaped tunnel and is located at its bottom. The catalytic mechanism proceeds in five steps, starting with the activation of the catalytic Cys via the deprotonation of its thiol group by the conserved active-site Glu residue, mediated using a water molecule. The activated thiol group of the catalytic Cys then attacks the electrophilic aldehyde group. This leads to the formation of a thiohemiacetal intermediate and hydride transfer to the pyridine ring of the coenzyme, yielding a thioester. The fourth step is represented by hydrolysis of the thioester and release of the product acid. Finally, the reduced cofactor NAD(P)H dissociates, and the enzyme is regenerated by binding a new molecule of the oxidized coenzyme [84].

**Figure 7:**
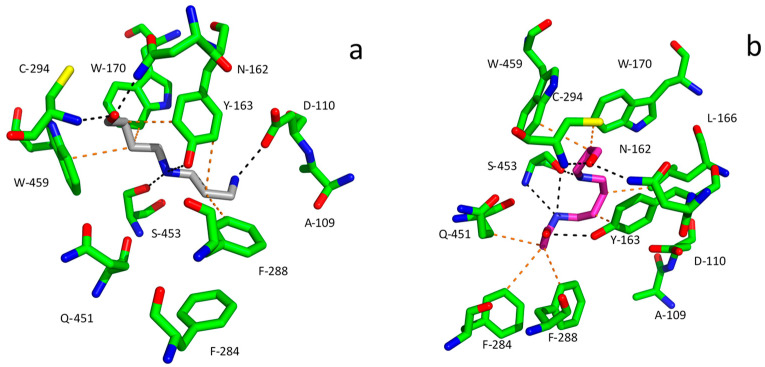
Molecular modeling of APBAL and BOPBD binding in the active site of PsAMADH1. The protein and ligand structures used were obtained from the PDB (https://www.rcsb.org, accessed on 18 September 2023) and ATB (https://atb.uq.edu.au, accessed on 18 September 2023) repositories, respectively, as PDF-formatted files. The depicted protein–ligand complexes were obtained using the Achilles Blind Docking server (https://bio-hpc.ucam.edu/achilles, accessed on 18 September 2023) [92] and processed for final images in PyMOL 1.3. Panel (**a**), PsAMADH1–APBAL complex; panel (**b**), PsAMADH1–BOPBD complex. The active site residues are shown in atom-coded colors (C—green, N—blue, O—red, S—yellow) and are labeled, the modeled hydrophobic and charge-based interactions of APBAL (carbons in grey) and BOPBD (carbons in magenta) are shown as orange and black dashes, respectively. This is the authors’ own work.

ALDHs with AMADH activity oxidize APAL to β-alanine (3-aminopropanoic acid) and ABAL to γ-aminobutyric acid (GABA). Both of these non-proteinogenic amino acids are involved in pathways of primary metabolism and participate in important physiological processes such as osmoprotection; β-alanine betaine and GABA belong to compatible osmolytes in plants [86,87]. Human ALDH9A1 oxidizes both APAL and ABAL well, but its best substrate is trimethylaminobutanal (TMABAL), as recently reported [88]. Pea seedling ALDH10 exists in two isoforms. TMABAL is the best substrate of isoenzyme 1 (PsAMADH1). It also oxidizes APAL well among other aminoaldehyde substrates, whereas the best substrate of isoenzyme 2 (PsAMADH2) is APAL itself. Both of these isoenzymes also oxidize ABAL well [83], which is in equilibrium with its cyclic form 1-pyrroline under the reaction conditions as already mentioned in this review. PsAMADH1 was shown to oxidize APBAL as a good substrate [27]. The entry of aldehydes into the substrate channel and their proper binding to the active site of ALDHs is mediated by the presence of specific amino acid residues, which are relevant for substrate specificity. The positively charged polyamine-derived aminoaldehydes are navigated by the presence of negatively charged residues at the channel entrance of AMADHs (Figure 7). For example, these are Asp110 and Asp113 in PsAMADH1 and 2 [89], from which the former is largely conserved among AMADHs [78]. The aromatic residues forming a hydrophobic pocket, e.g., Tyr163, Trp170, Trp288 and Trp459 in PsAMADH2, are responsible for the high affinity to APAL and ABAL. The highly conserved Tyr163 and Trp170 residues located in the central part of the substrate cavity are very important for anchoring the ω-aminoaldehyde substrate chain. The residues equivalent to Trp288 and Trp459 are variable, but in most cases, they retain the aromatic character [78]. AMADHs lacking an equivalent of Trp288 (Phe288 appears instead in PsAMADH1; Figure 7) prefer TMABAL as the best substrate [90]. The spatial orientation of Trp459 providing a sufficient distance from Tyr163 is important for binding the bulkier substrate betaine aldehyde (derived from choline) in specific AMADHs called betaine aldehyde dehydrogenases (BADHs; EC 1.2.1.8). This is achieved when no isoleucine but a smaller residue such as Ala or Cys (e.g., Ala441 in spinach BADH) is present in a position equivalent to Ile444 in PsAMADHs, which are known not to oxidize betaine aldehyde [90,91]. Figure 7 shows molecular modeling results for the binding of APBAL and BOPBD in the active site of PsAMADH1. The compounds appear in a favorable orientation of the aldehyde group towards the catalytic cysteine, and their binding is facilitated by interactions with many residues of the substrate cavity, such as Tyr163, Trp 170, Phe288 and Trp459. So far, only APBAL has been experimentally confirmed as a good substrate of the enzyme [27]; however, the present model shows that good substrate properties can also be expected for BOPBD.

## 7. Future Perspectives and Conclusions

The aminoaldehyde products of the enzymatic polyamine oxidation have been shown to be cytotoxic, although the concurrently generated hydrogen peroxide seems to be the major cytotoxic compound in the reaction mixtures (roughly 80% for human adenocarcinoma cells) as deduced from the moderating effect of catalase [93]. Regardless, the use of polyamine oxidation products to destroy cancer cells selectively and safely belongs to the ongoing experimental research directions on the road to innovative therapeutic strategies. Polyamine concentrations are high in growing tissues such as tumors (e.g., colorectal, breast and prostate cancer), which has been elucidated by their increased synthesis and uptake. When the production of polyamines in cancer cells is decreased by mutations or treatment, senescence and apoptosis occur. Polyamine metabolism is thus targeted with great interest in studies with anticancer compounds [13,94]. In this regard, it is very challenging that the oxidation of SPD and SPM by amine oxidases produces cytotoxic aminoaldehydes (releasing acrolein) and particularly hydrogen peroxide, which can be lethal to tumors when generated in situ. BSAO and SPM represent an enzymatic system, which has repeatedly been tested for this purpose [75,76,95]. Injections of BSAO into murine tumors (initiated by introducing B16 melanoma cells) for polyamine depletion led to a marked decrease in tumor growth. The enzyme was either used as a native protein or immobilized in hydrogel microbeads in the presence of serum albumin. The use of the immobilized enzyme was found to be advantageous compared with the native BSAO in two aspects. First, the tumor growth reduction was much higher (70 over 40%) at day 10, and a 70% apoptosis rate was achieved 120 h post treatment, which was much higher than the rate of <10% with the native enzyme. This was explained by the different kinetics of the release of cytotoxic products [96].

An interesting observation was achieved using LoVo human colon adenocarcinoma cells. Their multidrug-resistant variant LoVo DX (obtained by exposure to doxorubicin) was found to be more sensitive to the cytotoxic effect of the BSAO–SPM system than the wild type, which corresponded to the different mitochondrial morphology observed by transmission electron microscopy [93,95]. Severe mitochondrial damage was visible for the multidrug-resistant cells LoVo DX, particularly under localized hyperthermia conditions (42 °C). Thus, mitochondria appeared to have an important role in determining the differential responses between the two cell variants [94]. The effectiveness of anticancer treatment can be increased by combining multiple types of compounds, which are directed to different molecular targets and cellular processes. The PAO inactivator MDL 72527, i.e., *N*^1^,*N*^4^-*bis*(2,3-butadienyl)-1,4-butanediamine, which is also a lysosomotropic compound, sensitized LoVo cells to the toxicity induced by the presence of BSAO and SPM [95]. The use of MDL 72527 for the pretreatment (37 °C) was optimized at a concentration of 300 µM and an incubation time of 24 h. In consequence, the BSAO–SPM system was more cytotoxic (as deduced from a histochemical staining with methylene blue) and resistant cells were more sensitive [95]. Similar to BSAO and having a similar result, maize PAO was applied with exogenous SPM (12 μM and more) to treat wild-type LoVo and LoVo DX cells. In this case, however, hydrogen peroxide was the exclusive cytotoxic product of the reaction as demonstrated using catalase, which completely blocked the observed effect of the enzymatic system on cell viability [97]. This is not surprising, as the aminoaldehyde produced by maize PAO from SPM, i.e., APBAL, cyclizes to 1-(3-aminopropyl)pyrrolinium and does not release APAL and acrolein. Compared with BSAO, this enzyme is monomeric, shows a higher catalytic constant and is most active at pH 6.5, which better fits the environment in cancer cells [97].

The reaction products generated from SPD and SPM by maize PAO immobilized on hydroxyapatite were found to have anti-leishmanial effects in mice. As the effect was comparable with that of a synthetic ABAL, it was attributed to the produced aminoaldehydes [98]. Purified *Euphorbia characias* DAO was used in another study of the potential anti-leishmanial effects of diamine- and polyamine-derived aminoaldehydes (1–5 mM concentrations), wherein *Leishmania infantum* cells (promastigotes) were treated in vitro [99]. Whereas ABAL and 5-aminovaleraldehyde (deriving from the diamine cadaverine and cyclizing to 1-piperideine) showed rather stimulating effects, BOPBD (5 mM) had a strong inhibitory effect on the growth and viability of promastigotes [99].

In conclusion, the polyamine-derived aminoaldehydes, which spontaneously release acrolein upon decomposition (i.e., BOPBD and ABPAL), exhibit cytotoxic effects against human and animal cells. Acrolein is a powerful protein modifier similar to other aldehydes that are produced in the metabolism of lipids and sugars. Nucleophilic residues (Cys, Lys, His) are carbonylated by acrolein via the Michael reaction. Schiff bases of Lys or the secondary cyclic products FDP-Lys and MP-Lys are also formed as well as cross-links. These modifications are commonly analyzed using nanoLC-MS/MS and have been proposed as disease diagnostic markers (they appear, e.g., in atherosclerotic tissues). However, the main and still open challenge is to find clues for discovering clear and unambiguous linkages between aldehyde-induced modifications of specific proteins and the development of various diseases. It has been shown that the conjugation with acrolein can affect protein functions either positively or negatively. A recent review article summarizes that there are target proteins such as glyceraldehyde-3-phosphate dehydrogenase, cytoskeleton proteins (vimentin, actin, α- and β-tubulin) and apolipoprotein B-100 that are involved in cell viability and become inactivated by acrolein, while other proteins are activated, e.g., those involved in tissue damage processes (metalloproteinase-9, proheparanase). Cys, Lys and His were found the modified amino acids [100]. Regardless of the impact on activity, the modifications are accompanied by negative effects in the tissue. For example, modified cytoplasmic glyceraldehyde phosphate dehydrogenase is translocated to the nucleus, resulting in apoptosis. Interestingly, acrolein modifies the ability of immunoglobulins to recognize antigens, which leads to the aggravation of diseases. Acrolein is considered a serious cause of brain tissue damage, and it can be a principal cause of atherosclerosis [100]. Thus, its neutralization by scavenger compounds can alleviate the pathophysiology [58,59,60,61].

As modified peptides represent only a small fraction in the digests of modified proteins, further development of the enrichment procedures is highly desirable. The second aspect of the reactive aminoaldehydes concerns their potential therapeutic applications. It has been shown that the enzymatically oxidized SPD and SPM display anticancer and antiprotozoal effects, although hydrogen peroxide is the major cytotoxic product in the reaction mixture. BSAO, which oxidizes both of these polyamines, was tested in vivo in tumors as a therapeutic agent, mediating the release of the effector product molecules. The development of safe and effective anticancer compounds is highly desirable and will hopefully yield more promising outcomes for therapy in the future. Polyamine analogs and related compounds are being experimentally investigated as inhibitors for targeting polyamine transport and key enzymes of biosynthesis in cancer cells to reduce polyamine levels and induce apoptosis. They seem to be potentially useful in combined therapeutic strategies. Several bis(ethyl)-polyamine analogs have been developed, which induce the polyamine catabolic enzymes SSAT and SMO. As a result, polyamines are depleted with an increase in reactive oxygen species, leading to tumor-selective cytotoxicity. These effects were also elicited in combination with common chemotherapeutic compounds [15].

## Data Availability

Not applicable.

## Abbreviations

AcAPAL, 3-acetamidopropanal; AcPAO, *N*^1^-acetylpolyamine oxidase; AdoMet, *S*-adenosylmethione; AdoMet-DC, decarboxylated *S*-adenosylmethione; ABAL, 4-aminobutanal; ABPAL, *N*-(3-oxopropyl)-1,4-butanediamine i.e., 3-[(4-aminobutyl)amino]propanal; AGE, advanced glycoxidation/glycation end product; ADH, alcohol dehydrogenase; AKR, aldo/keto reductase; ALDH, aldehyde dehydrogenase; ALE, advanced lipoxidation end product; AMADH, aminoaldehyde dehydrogenase; APAL, 3-aminopropanal; APBAL, 4-[(3-aminopropyl)amino]butanal; BADH, betaine aldehyde dehydrogenase; BOPBD, *N*,*N*′-*bis*(3-oxopropyl)-1,4-butanediamine; BSA, bovine serum albumin; BSAO, bovine serum amine oxidase; DAO, diamine oxidase; DAP, propane-1,3-diamine; DNPH, 2,4-dinitrophenylhydrazine; EC, Enzyme Commission; ELISA, enzyme-linked immunosorbent assay; EMP-Lys, 5-ethyl-2-methylpyridinium-lysine; ESI, electrospray ionization; FDP-Lys, *N*^ε^-(formyl-3,4-dehydropiperidino)lysine; GABA, 4-aminobutyric acid; GC-MS, gas chromatography coupled with mass spectrometry; GSH, glutathione; HPLC, high-performance liquid chromatography; HPMA, 3-hydroxymercapturic acid; HSA, human serum albumin; IC_50_, 50% inhibitory concentration; MDL 72527, *N*^1^,*N*^4^-*bis*(2,3-butadienyl)-1,4-butanediamine; MP-Lys, *N*^ε^-methylpyridinium-lysine; *N*-2-MPG, *N*-(2-mercaptopropionyl)glycine; MS, mass spectrometry; MS/MS, tandem mass spectrometry; MTA, 5′-methylthioadenosine; MVK, methyl vinyl ketone; nanoLC, nanoflow liquid chromatography; NMR, nuclear magnetic resonance; NPC1, Niemann–Pick C1 protein; ODC, ornithine decarboxylase; PAO, polyamine oxidase; PsAMADH, pea (*Pisum sativum*) aminoaldehyde dehydrogenase; PUT, putrescine; Q-TOF, quadrupole time-of-flight; RP-HPLC, reversed-phase high-performance liquid chromatography; SAO, serum amine oxidase; SDS-PAGE, sodium dodecyl sulfate polyacrylamide gel electrophoresis; SMO, spermine oxidase; SPD, spermidine; SPDS, spermidine synthase; SPM, spermine; SPMS; spermine synthase; SSAT, spermidine/spermine *N*^1^-acetyltransferase; TLC, thin layer chromatography; TMABAL, trimethylaminobutanal.

## References

[1] LoPachin R.M., Gavin T. (2014). Molecular mechanisms of aldehyde toxicity: A chemical perspective. Chem. Res. Toxicol..

[2] Vistoli G., De Maddis D., Cipak A., Zarkovic N., Carini M., Aldini G. (2013). Advanced glycoxidation and lipoxidation end products (AGEs and ALEs): An overview of their mechanisms of formation. Free Rad. Res..

[3] Lai S.W.T., Lopez Gonzalez E.J., Zoukari T., Ki P., Shuck S.C. (2022). Methylglyoxal and its adducts: Induction, repair, and association with disease. Chem. Res. Toxicol..

[4] Domingues R.M., Domingues P., Melo T., Pérez-Sala D., Reis A., Spickett C.M. (2013). Lipoxidation adducts with peptides and proteins: Deleterious modifications or signaling mechanisms?. J. Proteom..

[5] Stevens J.F., Maier C.S. (2008). Acrolein: Sources, metabolism and biomolecular interactions relevant to human health and disease. Mol. Nutr. Food. Res..

[6] Guéraud F., Atalay M., Bresgen N., Cipak A., Eckl P.M., Huc L., Jouanin I., Siems W., Uchida K. (2010). Chemistry and biochemistry of lipid peroxidation products. Free Radic. Res..

[7] Uchida K., Kanematsu M., Sakai K., Matsuda T., Hattori N., Mizuno Y., Suzuki D., Miyata T., Noguchi N., Niki E. (1998). Protein-bound acrolein: Potential markers for oxidative stress. Proc. Natl. Acad. Sci. USA.

[8] Furuhata A., Ishii T., Kumazawa S., Yamada T., Nakayama T., Uchida K. (2003). *N*^ε^-(3-methylpyridinium)lysine, a major antigenic adduct generated in acrolein-modified protein. J. Biol. Chem..

[9] Suzuki Y.J., Carini M., Butterfield D.A. (2010). Protein carbonylation. Antioxid. Redox Signal..

[10] Baron K., Stasolla C. (2008). The role of polyamines during in vivo and in vitro development. In Vitro Cell. Dev. Biol.-Plant.

[11] Casero R.A., Pegg A.E. (2009). Polyamine catabolism and disease. Biochem. J..

[12] Chen D., Shao Q., Yin L., Younis A., Zheng B. (2019). Polyamine function in plants: Metabolism, regulation on development, and roles in abiotic stress responses. Front. Plant Sci..

[13] Sari I.N., Setiawan T., Kim K.S., Wijaya Y.T., Cho K.W., Kwon H.Y. (2021). Metabolism and function of polyamines in cancer progression. Cancer Lett..

[14] O’Brien P.J., Siraki A.G., Shangari N. (2005). Aldehyde sources, metabolism, molecular toxicity mechanisms, and possible effects on human health. Crit. Rev. Toxicol..

[15] Casero R.A., Stewart T.M., Pegg A.E. (2018). Polyamine metabolism and cancer: Treatments, challenges and opportunities. Nat. Rev. Cancer.

[16] Pietrangeli P., Federico R., Mondovì B., Morpurgo L. (2007). Substrate specificity of copper-containing plant amine oxidases. J. Inorg. Biochem..

[17] Pietrangeli P., Morpurgo L., Mondovì B., Di Paolo M.L., Rigo A., Floris G., Mondovì B. (2009). Soluble copper amine oxidases from mammals. Copper Amine Oxidases: Structures, Catalytic Mechanisms and Role in Pathophysiology.

[18] Tabor C.W., Tabor H., Bachrach U. (1964). Identification of the aminoaldehydes produced by the oxidation of spermine and spermidine with purified plasma amine oxidase. J. Biol. Chem..

[19] Kimes B.W., Morris D.R. (1971). Preparation and stability of oxidized polyamines. Biochim. Biophys. Acta.

[20] Seiler N., Knödgen B., Haegele K. (1982). *N*-(3-Aminopropyl)pyrrolidin-2-one, a product of spermidine catabolism in vivo. Biochem. J..

[21] Houen G., Bock K., Jensen A.L. (1994). HPLC and NMR investigation of the serum amine oxidase catalyzed oxidation of polyamines. Acta Chem. Scand..

[22] Quash G., Taylor D.R. (1970). Serum β-aminopropionaldehyde: Identification and origin. Clin. Chim. Acta.

[23] Seiler N. (2000). Oxidation of polyamines and brain injury. Neurochem. Res..

[24] Chae T.U., Kim W.J., Choi S., Park S.J., Lee S.Y. (2015). Metabolic engineering of *Escherichia coli* for the production of 1,3-diaminopropane, a three carbon diamine. Sci. Rep..

[25] Lee Y., Sayre L.M. (1998). Reaffirmation that metabolism of polyamines by bovine plasma amine oxidase occurs strictly at the primary amino termini. J. Biol. Chem..

[26] Houen G., Struve C., Søndergaard R., Friis T., Anthoni U., Nielsen P.H., Christophersen C., Petersen B.O., Duus J.Ø. (2005). Substrate specificity of the bovine serum amine oxidase and in situ characterization of aminoaldehydes by NMR spectroscopy. Biorg. Med. Chem..

[27] Šebela M., Brauner F., Radová A., Jacobsen S., Havliš J., Galuszka P., Peč P. (2000). Characterisation of a homogeneous plant aminoaldehyde dehydrogenase. Biochim. Biophys. Acta-Protein Struct. Molec. Enzym..

[28] Struve C., Christophersen C. (2003). Structural equilibrium and ring-chain tautomerism of aqueous solutions of 4-aminobutyraldehyde. Heterocycles.

[29] Šebela M., Radová A., Angelini R., Tavladoraki P., Frébort I., Peč P. (2001). FAD-containing polyamine oxidases: A timely challenge for researchers in biochemistry and physiology of plants. Plant Sci..

[30] Padiglia A., Medda R., Paci M., Sette M., Lorrai A., Floris G. (1997). Characterization of a cyclic compound fomed after spermine oxidation by lentil amine oxidase. Biochem. Mol. Biol. Int..

[31] Guo Z., Zhang Q., Zou H., Guo B., Ni J. (2002). A method for the analysis of low-mass molecules by maldi-tof mass spectrometry. Anal. Chem..

[32] Federico R., Ercolini L., Laurenzi M., Angelini R. (1996). Oxidation of acetylpolyamines by maize polyamine oxidase. Phytochemistry.

[33] Cervelli M., Amendola R., Polticelli F., Mariottini P. (2012). Spermine oxidase: Ten years after. Amino Acids.

[34] Moschou P.N., Sanmartin M., Andriopoulou A.H., Rojo E., Sanchez-Serrano J.J., Roubelakis-Angelakis K.A. (2008). Bridging the gap between plant and mammalian polyamine catabolism: A novel peroxisomal polyamine oxidase responsible for a full back-conversion pathway in *Arabidopsis*. Plant Physiol..

[35] Fincato P., Moschou P.N., Spedaletti V., Tavazza R., Angelini R., Federico R., Roubelakis-Angelakis K.A., Tavladoraki P. (2011). Functional diversity inside the *Arabidopsis* polyamine oxidase gene family. J. Exp. Bot..

[36] Häkkinen M.R., Hyvönen M.T., Auriola S., Casero R.A., Vepsäläinen J., Khomutov A.R., Alhonen L., Keinänen T.A. (2010). Metabolism of *N*-alkylated spermine analogues by polyamine and spermine oxidases. Amino Acids.

[37] Moriya S., Iwasaki K., Samejima K., Takao K., Kohda K., Hiramatsu K., Kawakita M. (2012). A mass spectrometric method to determine activities of enzymes involved in polyamine catabolism. Anal. Chim. Acta.

[38] Morgan D.M.L., Bachrach U., Assaraf Y.G., Harari E., Golenser J. (1986). The effect of purified aminoaldehydes produced by polyamine oxidation on the development in vitro of *Plasmodium falciparum* in normal and glucose-6-phosphate-dehydrogenase-deficient erythrocytes. Biochem. J..

[39] Hegre O.D., Marshall S., Hickey G.E. (1984). Spermidine cytotoxicity in vitro: Effect of serum and oxygen tension. In Vitro.

[40] Sharmin S., Sakata K., Kashiwagi K., Ueda S., Iwasaki S., Shirahata A., Igarashi K. (2001). Polyamine cytotoxicity in the presence of bovine serum amine oxidase. Biochem. Biophys. Res. Commun..

[41] Alarcon R.A. (1968). Fluorometric determination of acrolein and related compounds with *m*-aminophenol. Anal. Chem..

[42] Bonneau M.J., Poulin R. (2000). Spermine oxidation leads to necrosis with plasma membrane phosphatidylserine redistribution in mouse leukemia cells. Exp. Cell Res..

[43] Eisenberg T., Knauer H., Schauer A., Büttner S., Ruckenstuhl C., Carmona-Gutierrez D., Ring J., Schroeder S., Magnes C., Antonacci L. (2009). Induction of autophagy by spermidine promotes longevity. Nat. Cell Biol..

[44] Holbert C.E., Dunworth M., Foley J.R., Dunston T.T., Muray Stewart T., Casero R.A. (2020). Autophagy induction by exogenous polyamines is an artifact of bovine serum amine oxidase activity in culture serum. J. Biol. Chem..

[45] Sakata K., Kashiwagi K., Sharmin S., Ueda S., Igarashi K. (2003). Acrolein produced from polyamines as one of the uraemic toxins. Biochem. Soc. Trans..

[46] Tomitori H., Usui T., Saeki N., Ueda S., Kase H., Nishimura K., Kashiwagi K., Igarashi K. (2005). Polyamine oxidase and acrolein as novel biochemical markers of cerebral stroke. Stroke.

[47] Lambert C., McCue J., Portas M., Ouyang Y., Li J., Rosano T.G., Lazis A., Freed B.M. (2005). Acrolein in cigarette smoke inhibits T-cell responses. J. Allergy Clin. Immunol..

[48] Lambert C., Li J., Jonscher K., Yang T.C., Reigan P., Quintana M., Harvey J., Freed B.M. (2007). Acrolein inhibits cytokine gene expression by alkylating cysteine and arginine residues in the NF-κB1 DNA binding domain. J. Biol. Chem..

[49] Fanali G., di Masi A., Trezza V., Marino M., Fasano M., Ascenzi P. (2012). Human serum albumin: From bench to bedside. Mol. Aspects Med..

[50] Bein K., Birru R.L., Wells H., Larkin T.P., Cantrell P.S., Fagerburg M.V., Zeng X., Leikauf G.D. (2020). Albumin protects lung cells against acrolein cytotoxicity and acrolein-adducted albumin increases heme oxygenase 1 transcripts. Chem. Res. Toxicol..

[51] Seiner D.R., LaButti J.N., Gates K.S. (2007). Kinetics and mechanism of protein tyrosine phosphatase 1B inactivation by acrolein. Chem. Res. Toxicol..

[52] Gella A., Durany N. (2009). Oxidative stress in Alzheimer disease. Cell Adh. Migr..

[53] Pocernich C.B., Butterfield A.D. (2003). Acrolein inhibits NADH-linked mitochondrial enzyme activity: Implications for Alzheimer´s disease. Neurotox. Res..

[54] Fisher M. (2004). The ischemic penumbra: Identification, evolution and treatment concepts. Cerebrovasc. Dis..

[55] Li W., Yuan X.M., Ivanova S., Tracey K.J., Eaton J.W., Brunk U.T. (2003). 3-Aminopropanal, formed during cerebral ischaemia, is a potent lysosomotropic neurotoxin. Biochem. J..

[56] Yu Z., Li W., Hillman J., Brunk U.T. (2004). Human neuroblastoma (SH-SY5Y) cells are highly sensitive to the lysosomotrophic aldehyde 3-aminopropanal. Brain Res..

[57] Ivanova S., Botchkina G.I., Al-Abed Y., Meistrell M., Batliwalla F., Dubinsky J.M., Iadecola C., Wang H., Gregersen P.K., Eaton J.W. (1998). Cerebral ischemia enhances polyamine oxidation: Identification of enzymatically formed 3-aminopropanal as an endogenous mediator of neuronal and glial cell death. J. Exp. Med..

[58] Ivanova S., Batliwalla F., Mocco J., Kiss S., Huang J., Mack W., Coon A., Eaton J.W., Al-Abed Y., Gregersen P.K. (2002). Neuroprotection in cerebral ischemia by neutralization of 3-aminopropanal. Proc. Natl. Acad. Sci. USA.

[59] Wood P.L., Khan M.A., Moskal J.R., Todd K.C., Tanay V.A.M.I., Baker G. (2006). Aldehyde load in ischemia-reperfusion brain injury: Neuroprotection by neutralization of reactive aldehydes with phenelzine. Brain Res..

[60] Kim G.H., Kellner C.P., Hickman Z.L., Zacharia B.E., Starke R.M., Hwang B.Y., Ducruet A.F., Fernandez L., Mayer S.A., Tracey K.J. (2010). A phase I clinical trial of tiopronin, a putative neuroprotective agent, in aneurysmal subarachnoid hemorrhage. Neurosurgery.

[61] Ironside N., Christophe B., Bruce S., Carpenter A.M., Robison T., Yoh N., Cremers S., Landry D., Frey H.P., Chen C.J. (2020). A phase II randomized controlled trial of tiopronin for aneurysmal subarachnoid hemorrhage. J. Neurosurg..

[62] Yu Z., Li W., Brunk U.T. (2003). 3-Aminopropanal is a lysosomotropic aldehyde that causes oxidative stress and apoptosis by rupturing lysosomes. APMIS.

[63] Kaufmann A.M., Krise J.P. (2008). Niemann-Pick C1 functions in regulating lysosomal amine content. J. Biol. Chem..

[64] Wood P.L., Khan M.A., Moskal J.R. (2007). The concept of “aldehyde load” in neurodegenerative mechanisms: Cytotoxicity of the polyamine degradation products hydrogen peroxide, acrolein, 3-aminopropanal, 3-acetamidopropanal and 4-aminobutanal in a retinal ganglion cell line. Brain Res..

[65] Bellier J., Nokin M.J., Lardé E., Karoyan P., Peulen O., Castronovo V., Bellahcène A. (2019). Methylglyoxal, a potent inducer of AGEs, connects between diabetes and cancer. Diabetes Res. Clin. Pract..

[66] Ichihashi K., Osawa T., Toyokuni S., Uchida K. (2001). Endogenous formation of protein adducts with carcinogenic aldehydes. Implications for oxidative stress. J. Biol. Chem..

[67] Vasil’ev Y.V., Tzeng S.C., Huang L., Maier C.S. (2014). Protein modifications by electrophilic lipoxidation products: Adduct formation, chemical strategies and tandem mass spectrometry for their detection and identification. Mass Spectrom. Rev..

[68] Lo T.W.C., Westwood M.E., McLellan A.C., Selwood T., Thornalley P.J. (1994). Binding and modification of proteins by methylglyoxal under physiological conditions. A kinetic and mechanistic study with *N*^α^-acetylarginine, *N*^α^-acetylcysteine, and *N*^α^-acetyllysine and bovine serum albumin. J. Biol. Chem..

[69] Furuhata A., Nakamura M., Osawa T., Uchida K. (2002). Thiolation of protein-bound carcinogenic aldehyde. An electrophilic acrolein-lysine adduct that covalently binds to thiols. J. Biol. Chem..

[70] Chen H.J.C. (2023). Mass spectrometry analysis of DNA and protein adducts as biomarker in human exposure to cigarette smoking: Acrolein as an example. Chem. Res. Toxicol..

[71] Afonso C.B., Sousa B.C., Pitt A.R., Spickett C.M. (2018). A mass spectrometry approach for the identification and localization of small aldehyde modifications of proteins. Arch. Biochem. Biophys..

[72] Sousa B.C., Ahmed T., Dann W.L., Ashman J., Guy A., Durand T., Pitt A.R., Spickett C.M. (2019). Short-chain lipid peroxidation products form covalent adducts with pyruvate kinase and inhibit its activity in vitro and in breast cancer cells. Free Radic. Biol. Med..

[73] Kaminskas L.M., Pyke S.M., Burcham P.C. (2005). Differences in lysine adduction by acrolein and methyl vinyl ketone: Implications for cytotoxicity in cultured hepatocytes. Chem. Res. Toxicol..

[74] Cai J., Bhatnagar A., Pierce W.M. (2009). Protein modification by acrolein: Formation and stability of cysteine adducts. Chem. Res. Toxicol..

[75] Averill-Bates D.A., Agostinelli E., Przybytkowski E., Mondovì B. (1994). Aldehyde dehydrogenase and cytotoxicity of purified bovine serum amine oxidase and spermine in Chinese hamster ovary cells. Biochem. Cell Biol..

[76] Averill-Bates D.A., Ke Q., Tanel A., Roy J., Fortier G., Agostinelli E. (2008). Mechanism of cell death by spermine and amine oxidase in mouse melanoma cells. Int. J. Oncol..

[77] Brocker C., Vasiliou M., Carpenter S., Carpenter C., Zhang Y., Wang X., Kotchoni S.O., Wood A.J., Kirch H.H., Kopečný D. (2013). Aldehyde dehydrogenase (ALDH) superfamily in plants: Gene nomenclature and comparative genomics. Planta.

[78] Riveros-Rosas H., González-Segura L., Julián-Sánchez A., Díaz-Sánchez Á.G., Muñoz-Clares R.A. (2013). Structural determinants of substrate specificity in aldehyde dehydrogenases. Chem. Biol. Interact..

[79] Vasiliou V., Bairoch A., Tipton K.F., Nebert D.W. (1999). Eukaryotic aldehyde dehydrogenase (ALDH) genes: Human polymorphisms, and recommended nomenclature based on divergent evolution and chromosomal mapping. Pharmacogenetics.

[80] Jackson B., Brocker C., Thompson D.C., Black W., Vasiliou K., Nebert D.W., Vasilou V. (2011). Update on the aldehyde dehydrogenase gene (*ALDH*) superfamily. Hum. Genom..

[81] Riveros-Rosas H., Julián-Sánchez A., Moreno-Hagelsieb G., Muñoz-Clares R. (2019). Aldehyde dehydrogenase diversity in bacteria of the *Pseudomonas* genus. Chem. Biol. Interact..

[82] Ambroziak W., Kurys G., Pietruszko R. (1991). Aldehyde dehydrogenase (EC 1.2.1.3): Comparison of subcellular localization of the third isozyme that dehydrogenates γ-aminobutyraldehyde in rat, guinea pig and human liver. Comp. Biochem. Physiol..

[83] Tylichová M., Kopečný D., Moréra S., Briozzo P., Lenobel R., Snégaroff J., Šebela M. (2010). Structural and functional characterization of plant aminoaldehyde dehydrogenase from *Pisum sativum* with a broad specificity for natural and synthetic aminoaldehydes. J. Mol. Biol..

[84] Shortall K., Djeghader A., Magner E., Soulimane T. (2021). Insights into aldehyde dehydrogenase enzymes: A structural perspective. Front. Mol. Biosci..

[85] Liu Z.J., Sun Y.J., Rose J., Chung Y.J., Hsiao C.D., Chang W.R., Kuo I., Perozich J., Lindahl R., Hempel J. (1997). The first structure of an aldehyde dehydrogenase reveals novel interactions between NAD and the Rossmann fold. Nat. Struct. Biol..

[86] Cushman J.C. (2001). Osmoregulation in plants: Implications for agriculture. Am. Zool..

[87] Zarei A., Trobacher C.P., Shelp B.J. (2016). Arabidopsis aldehyde dehydrogenase 10 family members confer salt tolerance through putrescine-derived 4-aminobutyrate (GABA) production. Sci. Rep..

[88] Končitíková R., Vigouroux A., Kopečná M., Šebela M., Moréra S., Kopečný D. (2019). Kinetic and structural analysis of human ALDH9A1. Biosci. Rep..

[89] Kopečný D., Tylichová M., Snégaroff J., Popelková H., Šebela M. (2011). Carboxylate and aromatic active-site residues are determinants of high-affinity binding of ω-aminoaldehydes to plant aminoaldehyde dehydrogenases. FEBS J..

[90] Kopečný D., Končitíková R., Tylichová M., Vigouroux A., Moskalíková H., Soural M., Šebela M., Moréra S. (2013). Plant ALDH10 family: Identifying critical residues for substrate specificity and trapping a thiohemiacetal intermediate. J. Biol. Chem..

[91] Díaz-Sánchez Á.G., González-Segura L., Mújica-Jiménez C., Rudiño-Piñera E., Montiel C., Martínez-Castilla L.P., Muñoz-Clares R.A. (2012). Amino acid residues critical for the specificity for betaine aldehyde of the plant ALDH10 isoenzyme involved in the synthesis of glycine betaine. Plant Physiol..

[92] Sánchez-Linares I., Pérez-Sánchez H., Cecilia J.M., García J.M. (2012). High-throughput parallel blind virtual screening using BINDSURF. BMC Bioinform..

[93] Calcabrini A., Arancia G., Marra M., Crateri P., Befani O., Martone A., Agostinelli E. (2002). Enzymatic oxidation products of spermine induce greater cytotoxic effects on human multidrug- resistant colon carcinoma cells (LoVo) than on their wild type counterparts. Int. J. Cancer.

[94] Agostinelli E., Tempera G., Viceconte N., Saccoccio S., Battaglia V., Grancara S., Toninello A., Stevanato R. (2010). Potential anticancer application of polyamine oxidation products formed by amine oxidase: A new therapeutic approach. Amino Acids.

[95] Agostinelli E., Dalla Vedova L., Belli F., Condello M., Arancia G., Seiler N. (2006). Sensitization of human colon adenocarcinoma cells (LoVo) to reactive oxygen species by a lysosomotropic compound. Int. J. Oncol..

[96] Averill-Bates D.A., Chérif A., Agostinelli E., Tanel A., Fortier G. (2005). Anti-tumoral effect of native and immobilized bovine serum amine oxidase in a mouse melanoma model. Biochem. Pharmacol..

[97] Ohkubo S., Mancinelli R., Miglietta S., Cona A., Angelini R., Canettieri G., Spandidos D.A., Gaudio E., Agostinelli E. (2019). Maize polyamine oxidase in the presence of spermine/spermidine induces the apoptosis of LoVo human colon adenocarcinoma cells. Int. J. Oncol..

[98] Cona A., Federico R., Gramiccia M., Orsini S., Gradoni L. (1991). The amino aldehydes produced by spermine and spermidine oxidation with maize polyamine oxidase have anti-leishmanial effect. Biotechnol. Appl. Biochem..

[99] Massa S., Spanò D., Pintus F., Medda R., Floris G. (2010). Oxidation of di- and polyamines: In vitro effect of amino aldehydes on the vitality of *Leishmania promastigotes*. Med. Chem. Res..

[100] Kashiwagi K., Igarashi K. (2023). Molecular characteristics of toxicity of acrolein produced from spermine. Biomolecules.

